# Nutrigenetics and Nutrimiromics of the Circadian System: The Time for Human Health

**DOI:** 10.3390/ijms17030299

**Published:** 2016-02-26

**Authors:** Víctor Micó, Laura Díez-Ricote, Lidia Daimiel

**Affiliations:** Nutritional Genomics of Cardiovascular Disease and Obesity, IMDEA Food CEI UAM + CSIC, 28049 Madrid, Spain; victor.mico@imdea.org (V.M.); ldiezrico@ufl.edu (L.D.-R.)

**Keywords:** circadian-related genes, gene-diet interactions, personalized nutrition, circadian-microRNAs, single nucleotide polymorphisms, cardiovascular disease, type 2 diabetes mellitus, obesity

## Abstract

Even though the rhythmic oscillations of life have long been known, the precise molecular mechanisms of the biological clock are only recently being explored. Circadian rhythms are found in virtually all organisms and affect our lives. Thus, it is not surprising that the correct running of this clock is essential for cellular functions and health. The circadian system is composed of an intricate network of genes interwined in an intrincated transcriptional/translational feedback loop. The precise oscillation of this clock is controlled by the circadian genes that, in turn, regulate the circadian oscillations of many cellular pathways. Consequently, variations in these genes have been associated with human diseases and metabolic disorders. From a nutrigenetics point of view, some of these variations modify the individual response to the diet and interact with nutrients to modulate such response. This circadian feedback loop is also epigenetically modulated. Among the epigenetic mechanisms that control circadian rhythms, microRNAs are the least studied ones. In this paper, we review the variants of circadian-related genes associated to human disease and nutritional response and discuss the current knowledge about circadian microRNAs. Accumulated evidence on the genetics and epigenetics of the circadian system points to important implications of chronotherapy in the clinical practice, not only in terms of pharmacotherapy, but also for dietary interventions. However, interventional studies (especially nutritional trials) that include chronotherapy are scarce. Given the importance of chronobiology in human health such studies are warranted in the near future.

## 1. Introduction

Although the rhythmic oscillations of life have long been known, the molecular pieces that compose such a precise biological clock are slowly being revealed. Circadian rhythms exist in virtually all organisms, are integrated into our physiology and, thus, play important roles in the rhythm of our lives. Therefore, the circadian system is intimately linked to human health. Animal and human population studies have shown that circadian disruption is associated with diseases, such as metabolic syndrome (MetS) [1], type 2 diabetes mellitus (T2DM), obesity [2], cardiovascular disease (CVD) [3], and cancer [4]. Other studies have shown a relationship between chronobiology and success in weight-loss programs, suggesting that chronobiology should be taken into account in nutritional practice [5,6,7,8].

The relationship between circadian rhythm and cellular physiology has been extensively studied over the past decade and many relevant studies shed light on the regulation, as well as on the causes and consequences of circadian system misregulation. The circadian pathway has been described and it is currently being elucidated as new oscillating genes and new circadian posttranscriptional regulators are found. Many genes and metabolites oscillate following a circadian cycle and are regulated in a circadian manner [9,10,11].

As mentioned, circadian rhythms are associated with metabolic diseases such as T2DM, obesity, and MetS, all of them risk factors for CVD, the leading mortality cause worldwide. Thus, chronobiological characterization of these diseases is very important to define a chronobiological approach to treat them. Genetic and epigenetic characterization of the chronobiological system is particularly interesting, given that it is regulated by a transcriptional/translational feedback loop [12]. Some variants have been described in circadian-related genes that have been associated to disease risk. Moreover, some gene-diet interactions have been described for some of these variants. Additionally, several microRNAs modulate the circadian system and some of them are also modulated by diet.

Diet plays an important role in chronobiology and, in fact, fasting/feeding cycles constitute potent *zeitgebers* for peripheral clocks located in the gastrointestinal tract and the liver [13]. “Chrono-nutrition” is the research field that study the effect of time-restricted feeding on cellular physiology and metabolism. Conversely, nutritional genomics is the science that studies the relationship between genes and nutrients and how gene variants interact with nutrients and dietary patterns to modulate individual risks of disease. Nutritional genomics and chrono-nutrition have merged to study the relationship between gene variants in circadian-related genes and metabolic disorders and human health. This field also studies epigenetic mechanisms that govern the relationship between diet and metabolism. In this regard, recent works have focused on the study of dietary modulated microRNAs that regulate metabolism and are associated with metabolic disorders. In the context of chronobiology, nutritional genomics have been applied to the study of circadian-related microRNAs and their modulation by diet and dietary compounds. Nutritional genomic’s goal is to develop personalized nutritional approaches that take into account individual’s genetic and epigenetic information to design individualized dietary recommendations. Personalized nutrition combined with chrono-nutrition could contribute to the fight against obesity, T2DM, and CVD.

In this review, we summarize the state-of-the-art of the relationship between diet and chronobiology form a nutritional genomics perspective and discuss the potential of chronotherapy in personalized nutrition.

## 2. The Transcriptional/Translational Feedback Loop that Governs Circadian Rhythms

Circadian rhythms are governed by a central clock located in the supraquiasmatic nucleus (SCN) of the hypothalamus [3,14,15,16,17]. This central pacemaker receive inputs from the environment that serve as stimuli to synchronize its rhythms to the outside world. SCN uses these *zeitgebers* to synchronize peripheral clocks in other tissues such as liver, lungs, heart, stomach, or intestine [18]. Amongst these signals, light is the most powerful one. Light hits the retinal photoreceptors and the signal travels through the retinohypothalamic tract to the SCN. Temperature, fasting/eating, and rest/activity are others *zeitgebers* [14].

**Figure 1:**
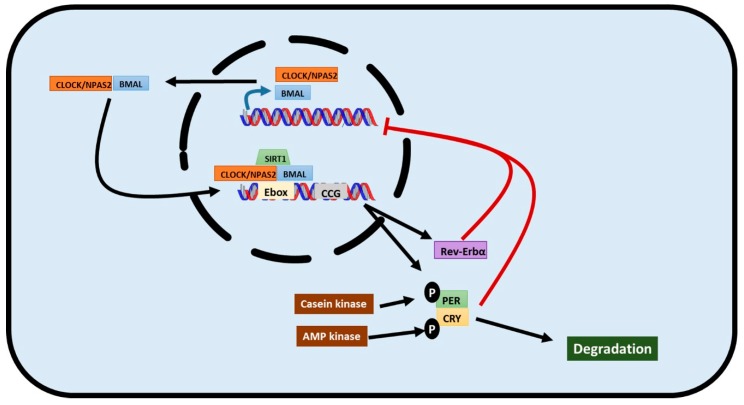
Graphical representation of the transcriptional/translational feedback loop that governs circadian rhythm. *CLOCK/BMAL1* heterodimer constitutes the first line of action. It binds to E-boxes in the promoter of target genes to activate them. Among its target genes, there are cryptochrome and period genes that form a heterodimer that negatively regulates *CLOCK/BMAL1* action in a negative feedback loop. *REV-ERB*α is also targeted by *CLOCK/BMAL1* and also negatively regulates its action. This network is also posttranscriptionally regulated through the phosphorylation-mediated degradation of cryptochrome and period genes.

Circadian rhythms are regulated by transcriptional/translational feedback loops in which the so-called clock genes play a key role [3,16,17] (Figure 1). Circadian locomotor output cycles kaput (*CLOCK*) or its alternative neuronal PAS domain protein 2 (*NPAS2*) and brain and muscle ARNT-like protein 1 (*BMAL1*), are the core clock genes which trigger the signaling pathway. *CLOCK* and *BMAL1* dimerize in the cytosol and are translocated to the nucleus where they bind to E-box sequences in the promoter regions of several clock genes stimulating their expression. Thus, period genes (*PER1*, *PER2*, and *PER3*), cryptochrome genes (*CRY1* and *CRY2*) reverse erythroblastosis virus α gene (*REV-ERB*α), retinoid related orphan receptor α gene (*ROR*α), and other clock-controlled genes (CCG) are expressed. *CLOCK* and *ROR*α also induce *BMAL1* expression. Conversely, there is a feedback inhibition: period and cryptochrome genes dimerize in the cytosol, translocate into the nucleus, and inhibit *BMAL1* expression. *REV-ERB*α also participates in *BMAL1* inhibition [3,16,17].

Clock genes are also posttranscriptionally regulated. Casein Kinase phosphorylates *PER*, targeting it for degradation via ubiquitin complex [19,20]. *CRY* is phosphorylated by AMP kinase and it is degraded by proteasome 26s [19]. Sirtuin 1 (*SIRT1*) is a histone deacetylase that interacts with *CLOCK* and *BMAL1* when they bind to the promoter sequence of clock-controlled genes, allowing rhythmic expression of proteins in the liver and synchronizing cellular metabolism with circadian rhythms [20]. *CLOCK* is also a histone acetyltransferase that acetylates *BMAL1* and *HDAC*, a histone deacetylase which, in conjunction with *REV-ERB*α, regulate circadian lipid metabolism [19].

## 3. Peripheral Clocks, Time of Feeding and Metabolic Consequences of Chronodisruption

Clock genes are not only expressed in the SCN, but also in peripheral tissues such as heart, liver, adipose tissue, and muscle, which act as peripheral clocks. These are metabolic tissues; therefore, circadian genes are involved in metabolism regulation [13]. Several studies have showed circadian regulation of many metabolic genes. For instance, nuclear receptors, such as peroxisome proliferator-activated receptor (PPAR) family, exhibit circadian expression. PPARs are activated by ω-3 polyunsturaed fatty acids (PUFAs) and *CLOCK-BMAL1* dimer through an E-box sequence in their promoters. They play a role in energy homeostasis and lipid metabolism [21,22]. They can also modulate *PER2* activity [22]. Sterol regulatory element binding protein 1 family (*SREBP1*) controls lipid synthesis enzymes like fatty acid synthase (*FAS*), acetyl-CoA carboxylase 1 (*ACC1*), and HMG-CoA reductase. These are expressed in a circadian fashion, peaking at the end of the rest period and at the beginning of the active period, correlating with *PPAR*α expression peak. However, in *PPAR*α null mice these cycles are not observed [22,23]. Β-oxidation enzymes, such as carnitine palmitoyltransferase (*CPT1*), hormone sensitive lipase (*HSL*), adipose triglyceride lipase (*ATGL*), medium chain acyl-CoA dehydrogenase (*MCAD*), and diacylglycerol acyltransferase 2 (*DGAT2*) are circadianly regulated [24]. Regarding glucose homeostasis, carbohydrates metabolic genes are also regulated in a circadian manner. For instance, glycogen synthase peaks during the active period and glycogen phosphorylase does it during the resting period. In *Clock* mutant mice, these cycles are diminished. In addition, phosphoenolpyruvate carboxykinase (*PEPCK*) is regulated by *BMAL1* and peaks during the beginning of the active period [24]. These findings explain why chronodisruption affects human metabolism leading to metabolic disoders.

The peripheral clocks rhythmicity is independent of that of the SCN [25]; however, both must be synchronized to maintain cellular physiology rhythms. Chronodisruption occurs when these clocks are desynchronized, possibly leading to disease. Chronodisruption can be defined as a relevant disturbance of the circadian organization of physiology, endocrinology, metabolism, and behavior [26] that has been associated with the development of disease. In a study in which mice tissues were collected over the period of 48 h, during which mice were kept in total darkness, most of the drug target genes, metabolic genes, and genes involved in diseases overlapped with circadian genes [11]. The MetS is more prevalent [27]; resistin levels, an atherosclerosis biomarker, are increased [28], and myocardial infarction risk is higher [29] among shift-workers. “Social” jet lag, which is the disruption between social and circadian rhythms, and sleep disturbances, can lead to MetS and CVD [30,31]. Some examples of social chronodisruption are shift-work and jet-lag. Shift work, especially at night, is associated to differences in melatonin expression and metabolic and physiologic disorders [32], including, but not limited to, alterations in triglycerides and cholesterol levels, uric acid, and blood pressure. [33]. Jet-lag seems to have the same effects as shift-work and is also associated with obesity, hypertension, and MetS [34]. The mechanisms underlying these associations are poorly understood but impairment of leptin and insuline signaling probably contribute to the chronodisruption-associated metabolic disturbancies [35,36]. This suggests that chronotherapy should be considered as integral part of the treatment for these diseases.

Apart from the role of light as an external input for circadian entry, meal schedules, and activity/rest cycles are effective *zeitebergs* for peripheral clocks but not for the central one [18]. A misalignment with the SCN can occur when meal schedules are shifted, leading to metabolic diseases [18]. In an experiment in which mice were fed during their rest period to emulate shift-workers, the animals showed higher glucose, triglyceride, and free fatty acid blood levels and higher hepatic cholesterol, triglyceride, and low-density lipoprotein (LDL) levels, compared to control normally fed mice, resembling MetS symptoms. Moreover, gene expression was shifted. Genes which usually were expressed during the active period were expressed during the rest period and rest period genes were expressed during active period [37]. In addition, mice fed during their rest period tend to gain more weight than mice fed during their active period [8]. Other studies showed the importance of breakfast to synchronize peripheral tissues with the central clock. In this study, rats were deprived of breakfast or dinner and they were compared to those that had three meals/day. Mice that did not have breakfast showed a delayed phase in the hepatic clock. Mice that did not have dinner showed less weight gain, less adipogenesis and fatty acid synthase activity and lower glucose and triglyceride levels [38].

Specific nutrients can also alter clock-controlled genes expression. Glucose can regulate *BMAL1* and period expression [39] and mice fed a high-fat diet showed altered lipid metabolism gene expression [13]. A study carried out by Eckel-Mahn *et al.* showed that most liver metabolites oscillate following a circadian rhythm and that such oscillation is regulated by the clock transcriptome and eating/fasting cycles and contribute to maintain liver homeostasis [9].

Human studies have also highlighted the key role of the circadian system in human health. Circadian biomarkers in obese patients have been analyzed to assess chronobiological expression of these parameters in MetS. Wrist temperature, sleep onset and offset, and salivary morning cortisol are the main circadian biomarkers associated with MetS because chronobiological alteration of these biomarkers have been associated to a higher risk of MetS [5]. Rhythmic feeding synchronizes peripheral clocks, thus when the feeding pattern is modified, metabolic complications appear. A clinical trial showed that regardless age, gender, MetS characteristics of the patient, level of activity, and sleeping patterns, subjects who had their main meal later in the day (late eaters) lost less weight than early eaters even though they consumed the same amount of calories per day. Moreover, late eaters showed higher insulin levels [6]. In another study late eaters showed less resting energy expenditure, less carbohydrate oxidation, less glucose tolerance and altered daily variations of cortisol, and wrist temperature leading to less circadian signaling to peripheral clocks [40]. Taken together, these data suggest that meal time has a substantial effect on metabolic genes regulation and that SCN cycles and peripheral clocks disruption due to altered fasting/feeding cycles could lead to T2DM and MetS and increased CVD risk.

## 4. Gene Variants and Gene-Diet Interactions in Circadian-Related Genes Associated to Disease

Some gene variants can predispose individuals to different diseases. That is the case of the *FTO* (fat mass and obesity associated) gene and its association with obesity and related traits [41,42,43] or the *TCF7L2* gene and its association with T2DM [44,45,46]. Moreover, some of the well-known disease-related gene variants interact with diet and dietary compounds to modulate such predisposition [47,48]. Other gene variants modulate the individual response to a weight-loss program in terms of reduction of weight, body mass index (BMI), or waist circumference [49,50]. Given the role that chronobiology plays in human health and given that the circadian system is composed of an intricate gene network that controls oscillating gene expression, it has been hypothesized that circadian-related gene variants could be associated with different diseases. For that reason, nutritional genomics studies recently focused on the interactions between circadian-related genes and nutrients to modulate disease risk and individual variability in weight-loss programs.

### 4.1. Lessons from Animal Models of Genetic Chronodisruption

Animal studies with mutations in different circadian-related genes have shown that circadian rhythm disruption leads to abnormal glucose and lipid metabolism and to the development of associated phenotypes such as obesity and T2DM. The most studied model is *Clock* gene disruption in mice carrying a deletion in exon 19 in this gene. These mice develop obesity and some traits associated to MetS. They also showed alterations in the normal diurnal feeding rhythm and energy regulation [51]. Another metabolic characteristic of these *Clock* mutant mice was hyperglycemia and hypoinsulinemia, a suggestive pattern of a defect in the insulin signaling pathway [51]. *Bmal1* disruption also led to β-cell dysfunction and, consequently, to impaired glucose tolerance, reduced insulin secretion, and diabetes [52]. In addition, these mutant mice showed impaired gluconeogenesis due to impaired conversion of pyruvate to glucose and changes in the hepatic phosphoenolpyruvate carboxykinase enzyme activity [53]. It has also been described that *Clock* and *Bmal1* mutants have a dysfunction in leptin metabolism that contribute to explain the obesogenic phenotype associated to these mutations [54]. Additionally, *Clock* disruption exacerbated the obesogenic phenotype of ob/ob mice [55] and the atherogenic phenotype of *ApoE* null mice [56]. In a recently-developed cardiomyocyte-specific *Clock* mutant mouse model, Peliciari-Garcia *et al.* showed that there was a different metabolic adaptation in these mutant hearts compared with wild type in response to a streptozotocin-induced diabetes [57].

*Per1* mutant mice exhibited higher food intake but lower body weight and increased glucose metabolism [58]. Conversely, *Per2* mutant mice are obese although the food intake is not higher than in wild-type mice [59]. These mutant mice have glucocorticoid rhythm and diurnal appetite control affected. Sleep restriction induces a transcriptional reprogramming of white adipose tissue leading to increased lipogenesis, secretion of leptin and food intake, all of them hallmarks of obesity and associated leptin resistance. However, double *Per1*/*Per2* mutants seem to be protected from sleep restriction effects [60].

*Rev-erb*α knockout mice showed a phase-shift in the expression of genes involved in lipid metabolism, leading to dysregulation of hepatic cholesterol and bile acid metabolism [61]. These knockout mice have impaired Srebp-mediated cholesterol metabolism due to impaired inhibition of *Insig2* by *Rev-erb*α. Conversely, mice deficient in *Cry1* showed resistance to high-fat diet-induced obesity, despite a similar caloric intake than wild-type mice fed the same diet [62].

### 4.2. Gene Variants in Circadian-RelatedG Are Associated with Metabolic Disorders and Modulate the Individual Response to Diet: Human Population Studies

The application of nutritional genomics to chronobiological studies is teaching us how gene variants in circadian-related genes may increase the risk of metabolic disorders and related diseases such as obesity, T2DM, or CVD. Circadian-related gene variants are also associated with CVD traits like hypertension (Table 1). Dashti *et al*. analyzed the association of 5004 single nucleotide polymorphisms (SNPs) in 18 circadian-related genes with blood pressure and found that although these SNPs did not contribute to the individual variance in the diastolic blood pressure, they collectively explained 7.1% of the variance in systolic blood pressure in Genetics of Lipid Lowering Drugs and Diet Network population (GOLDN) and Boston Puerto Rican Health Study Population (BPRHS) populations [63]. Moreover, some of the reported associations between variants in circadian-related genes and metabolic disorders can be modulated by diet.

#### 4.2.1. Variants in the *CLOCK* Gene

Most studies have focused on SNPs in the *CLOCK* gene (Table 1). SNPs rs3749474 and rs1801260 located in the 3′-UTR were associated with weight and BMI as carriers of the minor alleles showed higher BMI and weight than non-carriers [64]. The reported association between rs3749474 and obesity-related traits may be partly due to a higher energy intake observed in carriers of the minor allele [65]. The SNP rs1801260 was also associated with the individual response to a weight-loss program since obese subjects with the minor allele were less successful losing weight than obese patients homozygous for the common allele, especially if they were classified as “emotional eaters” [66,67]. This difference in the response to a weight-loss program could be due to differences in the sleep duration of the patients since subjects carrying the minor allele were more prone to sleep ≤6 hours/day than non-carriers [64]. It has been previously described that sleep duration is associated with obesity, T2DM, and CVD, probably as a consequence of changes in dietary intake [68]. The effect of the rs1801260 SNP on weight loss were particularly evident in older individuals. This SNP was also associated with higher ghrelin plasma levels and with circadian abnormalities explained by a more evening chronotype and less-stable circadian rhythms [66,69]. Ghrelin, the so-called “hunger hormone”, is a neuropeptide secreted by the stomach during fasting conditions and acts on the hypothalamus to induce food intake [70]. Minor allele carriers also showed higher waist circumference. This association is modulated by saturated fatty acids (SFA) intake, as when SFA intake was low, this association disappeared [71]. Interestingly, other *CLOCK* rs1801260 SNP gene-diet interactions have been described. For instance, in patients with MetS from the CORDIOPREV study it has been shown that homozygote carriers of the major allele displayed lower plasma insulin levels, lower HOMA-IR (homesotatic model assesment-insulin resistance) and higher insulin sensitivity in response to a low-fat diet than carriers of the minor allele [72]. Interaction analyses in 722 participants of the Boston-Puerto Rican Health Study showed that the minor C allele was protective against high LDL-cholesterol plasma levels in the high SFA intake group [73]. rs1801260 SNP has also been associated with higher risk of prevalent diabetes in a Japanese population, especially in lean subjects [74]. rs4580704 SNP located in intron 9 has been associated with BMI. Minor allele carriers showed lower weight and BMI and had 31% lower risk of T2DM than non-carriers. They also showed lower blood pressure and, consequently a 45% lower risk of hypertension. However, this study did not find an association with fasting levels of triglycerides, high-density lipoprotein (HDL) cholesterol, LDL cholesterol, and total cholesterol, although minor allele carriers showed lower triglyceridemia after a fat-loading test than non-carriers [71]. A gene-diet interaction was found for the association between SNP rs4580704 and diabetes-related parameters (fasting glucose and HOMA). In this regard, the association with lower plasma glucose levels and HOMA in minor allele carriers was significant only when monounsaturated fatty acids (MUFA) intake was high [71]. Protection conferred by the minor allele against obesity, hypertension, and T2DM could be partly explained by lower energy intake observed in carriers of that allele [65]. These results come from observational studies belonging to the GOLDN population. However, while this manuscript was being prepared Corella *et al.* reported an interesting interaction between this SNP and the Mediterranean Diet in the interventional trial PREDIMED [75]. They corroborated that homozygote carriers of the G allele showed lower weight, BMI and waist circumference, as well as lower fasting glucose levels in non-diabetic subjects. Carriers of the G allele, both homozygotes and heterozygotes, showed a slightly higher adherence to the Mediterranean Diet at baseline. According to these associations, they found that G-carriers had lower risk of T2DM. Interestingly, this protective effect was stronger and more significant in the group treated with Mediterranean Diet. Diabetic G-carriers also showed lower risk of CVD, however, apparently this protective effect was not exacerbated by the Mediterranean Diet [75]. Together, these studies highlight the relationship between *CLOCK* variants and T2DM and obesity-related traits, but they also point to the diet as potential modulator of this relationship and suggest that *CLOCK* variants should be included in SNP panels for personalized nutrition.

#### 4.2.2. Variants in Other Circadian-Related Genes

Cryptochrome and period genes, as well as *REV-ERB*α*1*, constitute the second key components of the circadian system, responsible for the negative feedback loop that controls *CLOCK/BMAL1* expression and have been also associated with metabolic and glycemic traits related to obesity and T2DM. Cryptochrome genes have been recently associated with T2DM and related traits and with MetS and CVD [76,77,78,79] (Table 1). *CRY1* rs2287161 located 3′ downstream of *CRY1* has been associates with HOMA-IR, although this association was modulated by carbohydrate intake, since homozygous subjects for the minor C allele showed higher HOMA-IR when carbohydrate intake was higher than 41.65% of total energy intake, but not when carbohydrate intake was lower [76]. *CRY2* rs11605929 SNP has been associated with fasting glucose levels and T2DM risk in an Asian population [77]. Protective effect of this *CRY2* SNP against T2DM could be explained by better β-cell function [80] and changes in energy expenditure in response to fat content of a weight-loss diet [81]. However, a recent meta-analysis could not replicate this previous association between *CRY2* rs11605924 SNP and fasting glucose [78]. Another study combining data from the Punjabi, DIAGRAM, and SAT2D cohorts found that *CRY2* rs2292912 SNP and rs12315175 SNP near *CRY1* were associated with higher and lower risk of T2DM, respectively. Nevertheless, we must consider that these associations were nominal and lost statistical significance when authors applied Bonferroni’s correction [82] (Table 1).

Polymorphisms in period genes have been associated with circadian and behavioral alterations. These alterations may play a role in the observed association between common variants in *PER1* gene and extreme obesity [83] and between rs2304672 and rs4663302 SNPs in *PER2* and abdominal obesity [84,85] (Table 1). Additionally, Kelly *et al.* showed that rs7602358 G allele near *PER2* was negatively associated with T2DM, while the *BMAL1* rs11022775 T allele was associated with an increased risk of disease in the Punjabi cohort. However, neither of these associations was replicated in SAT2D or DIAGRAM datasets [82]. rs6486121 and rs7950226 SNPs in *BMAL1* have also been associated with hypertension and T2DM, respectively in 1304 individuals from 424 British T2D families from the Diabetes in Families (DIF) study collection. However, these associations were not significant after correction for multiple testing [86]. rs6486121, rs3789327, and rs969485 CCA haplotypes were significantly associated with hypertension [86] (Table 1).

Three recent studies have showed an association between variants in the *REV-ERB*α gene and obesity (Table 1). A study performed by Garaulet *et al.* in 2014 showed that rs2314339 in *REV-ERB*α*1* is associated with abdominal obesity in two populations of European origin (Mediterranean and North American). Minor allele carriers had lower waist circumference and BMI and were less prone to have abdominal obesity. This associations were modulated by MUFA intake in the Mediterranean population (which showed higher MUFA intake) since lower BMI associated to the minor allele was significant only when MUFA intake was ≥ 55% of total fat [87]. Ruano *et al.* showed that homozygote carriers of the A allele of rs939347 were prone to obesity. However, this association was significant only in men [88]. Finally, Goumidi *et al.* showed that the minor T allele of rs2071427 was associated with higher BMI in adults and adolescents [89].

In human populations, genome-wide association studies have revealed associations between circadian clock–related gene variants of *MTNR1B* and risk of obesity and T2DM (Table 1). Bouatia-Naji *et al.* found that the rs1387153 T allele was associated with increased fasting glucose levels and risk of developing hyperglycemia or T2DM [90]. Lyssenko *et al.* found a similar association with rs10830963 SNP allele risk. They also found decreased insulin secretion in risk allele carriers [91]. These results were corroborated by Prokopenko *et al.* in a GWAS involving 36,610 individuals of European origin [92] and by Zheng *et al.* in Caucasian and Hispanic children and adolescents [93]. A recent meta-analysis including 28,190 individuals of European descendent from 15 cohort studies belonging to the CHARGE consortium replicated previously-reported associations between *MTNR1B* variants and glycemic traits [78]. This study also found some gene-diet interactions. For instance, an interaction was found for carbohydrate intake and sleep duration with rs1387153 that modulated fasting glucose and BMI. However, theses interactions did not reach statistical significance. This SNP also interacted with dietary fat to modulate respiratory quotient in obese individuals under a weight-loss program [81]. Another meta-analysis also found and association between rs10830963 and T2DM risk in Caucasians but not in Asians [94]. These results are in concordance with those reported by Been *et al.*, who failed to find any association between SNPs in *MTNR1B* and glycemic traits in Sikhs from India, although they did find an association between rs1374645 with lower fasting glucose in the group with the lowest BMI [95]. *MTNR1B* encodes melatonin receptor 1B. Melatonin is a pineal hormone synthesized and released with a robust daily oscillation that is regulated by the master circadian clock and ambient light exposure in the SCN [96]. The association between these variants in MTNR1B gene and T2DM and related traits could be mediated by a β-cell dysfunction that leads to impaired insulin secretion [91,97]. Moreover, a recent report has suggested a mechanism that partially explains the observed association between rs10830963 and the risk of T2DM. Such association could be mediated by an increase of *FOXA2*-enhancer activity that leads to higher *MTNR1B* expression in β-cells and liver cells in risk allele carriers [98] (Table 1).

These studies extend our previous knowledge about *CLOCK* variants and obesity and T2DM to other circadian-related genes. However, in most cases these differences were not statistically significant or failed to find an association in non-caucasian populations. Thus, these results need further validation before the inclusion of these SNPs in personalyzed nutrition strategies. Additionally, ethnicity is an important issue that suggests that personalized nutrition strategies should take ethnic backgrounds into account.

## 5. Nutrimiromics of the Circadian System

Genes related to circadian rhythm are regulated by epigenetic processes. Epigenetics refers to gene expression modification due to external stimuli without alteration of the underlying gene sequence [99]. One recently discovered epigenetic mechanisms that regulate gene expression is through microRNAs. MicroRNAs are small non-coding RNA sequences of 22–24 nucleotides located in intra- or inter-regions of protein coding genes [100] that act as specific inhibitors of their target genes [101]. MicroRNAs are transcribed by RNA polymerase II generating a precursor hairpin-structured sequence called primary microRNA or pri-microRNA [102]. Then, a microprocessor complex composed by Drosha and Dgcr8 processes the pri-miRNA to generate a short hairpin sequence of approximately 65 nucleotides named precursor microRNA or pre-miRNA. Pre-miRNA is then exported to the cytoplasm via Exportin 5 (Exp-5), where it is processed by Dicer to form a 22–25 nucleotides duplex. Finally, this duplex sequence splits into two single mature sequences that are associated with Argonaute protein (AGO) forming an RNA-induced silencing complex (RISC), which acts on target genes. MicroRNAs recognize specific target sequences in the 3′-UTR of their target genes and guide RISC complex to mRNA. MicroRNAs inhibit mRNA expression by promoting mRNA degradation or by inhibiting protein translation [102,103].

MicroRNAs are important modulators of gene expression that control many cellular and physiological processes [104] and some of them have been associated with diseases such as cancer [105], T2DM , atherosclerosis, or dyslipidemia [106,107]. The role of microRNAs in human disease is linked to their involvement in many physiological process like cholesterol metabolism [108], insulin signaling [109], inflammation, and endothelial function [107], and recent reports have shown their influence in circadian rhythm [110]. MicroRNAs have also been recognized as potential biomarkers of disease onset and progression as they are present in plasma and other biofluids like urine or cerebrospinal fluid. In the blood stream, microRNAs can be associated to lipoproteins, exosomes, and protein complexes that prevent them from degradation by RNases [111].

### 5.1. Circadian Expression of MicroRNAs

**Figure 2:**
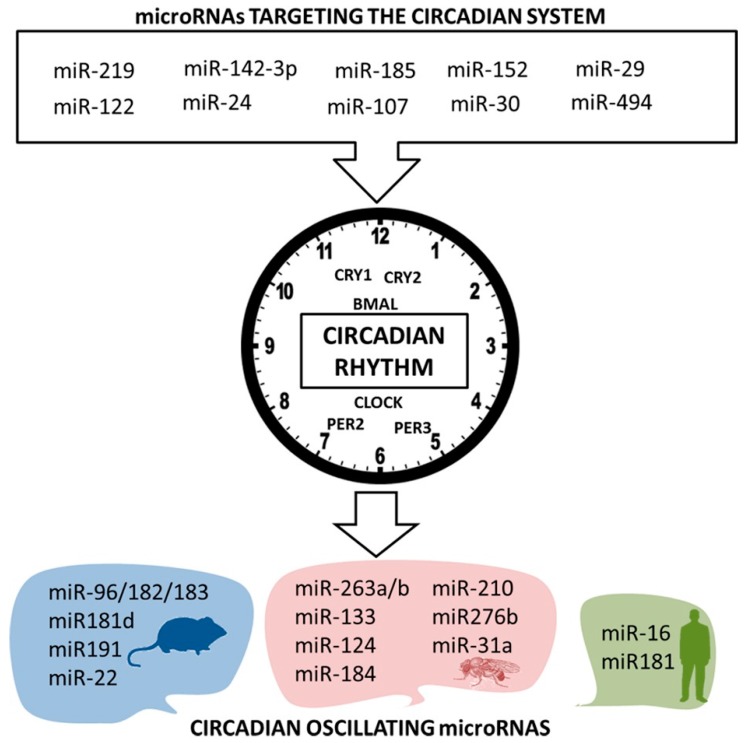
Circadian microRNAs. Some microRNAs involved in human disease are expressed in a circadian manner. Otherwise, some microRNAs involved in human disease regulate circadian core genes.

Like hormones, genes, proteins, and metabolites, many microRNAs oscillate in a circadian manner [8,10] (Figure 2). A notable example is the microRNA cluster composed by miR-96/miR-182/miR-183, which in a murine model exhibits diurnal variation and is implicated in melatonin production in the pineal gland [112]. In addition to this effect, upregulation of this cluster is implicated in hepatocellular carcinoma and breast cancer [113,114]. Additionally, Kinoshita *et al.* showed the importance of rhythmic oscillations of miR-96-5p in the regulation of glutathione levels via excitatory amino acid carrier 1 (*EAAC1*) that have a protective role in the brain [115].

Other experiments in animal models have contributed to decipher the rhythm of the microRNAs-mediated regulation. Na *et al.* identified different pairs of hepatic miRNA-mRNA targets with circadian expression. Specifically, they detected 24 microRNAs and 10 clock genes [116] that oscillated following a circadian cycle. The authors hypothesized that the identification of these oscillating microRNAs–mRNAs pairs could provide a better understanding of critical genes involved in circadian rhythm in a still unexplored field of study. More relevant microRNAs detected were miR-181d and miR-191, implicated in circadian transcription factor regulation in mouse liver, suggesting the importance of the cyclic expression of these microRNAs for the cyclic regulation of the expression of clock genes like *CLOCK* and *BMAL1* in the liver [116]. miR-191 has been linked to several cancers and other diseases like T2DM, Crohn’s disease, pulmonary hypertension, and Alzheimer’s disease [117]. Additionally, circulating miR-191 levels are increased in patients with coronary artery calcification [118] but are not modified in patients with T2DM [119]. Whether other tissues presented circadian microRNAs was unknown until the recent study carried out by Zhang *et al.* who analyzed the circadian expression of genes and non-coding RNAs in 12 mouse tissues. They found that levels of 39 microRNAs oscillated and that these oscillations were opposite to that of their target genes. Among these microRNAs, they highlighted miR-22 and its predicted target *Ptgs1*. *Ptgs1* is the primary target of aspirin and authors suggested that the circadian regulation of this gene by miR-22 could explain the observed rhythms in aspirin’s cardioprotective effects [11].

Using Drophosila melanogaster as animal model, Yang *et al.* checked for the presence of several microRNAs with a circadian expression pattern in contrast to cyc01 mutant models where the circadian cycle is altered. In this experiment, the authors observed that two microRNAs, dme-miR-263a and dme-miR-263b, had a strong circadian regulation, while they were unaltered in the mutant model. Furthermore, these microRNAs also had predicted target genes implicated in period phosphorylation, so these circadian regulated microRNAs where also circadian regulators. Other microRNAs with a different expression pattern in wild-type *versus* mutant flies were miR-133, miR-124, miR-184, miR-210, miR-276b, and miR-31a, microRNAs that could target clock genes such as *Clk*, *Per*, *Dbt*, *Tws*, and *Slo* as predicted by *in silico* analyses [120].

All these experiments in animal models suggest that human microRNAs are also regulated by the circadian system. However, little evidence exists in human. Figueredo *et al.* have recently demonstrated daily variation in miR-16 and miR-181 expression in human leukocytes, both microRNAs peaked between 8:00 a.m. and 16:00 p.m. [121]. miR-181 has been associated with glioblastoma [122] and it has been proposed to be a modulator of the lipid droplet content in human hepatic cells [123], suggesting a link with lipid metabolism. Accordingly, circulating levels of miR-181 have been suggested to be a potential biomarker of non-alcoholic fatty liver disease [124], and have been found to be decreased in monocytes of obese subjects, although weight loss normalized its expression [125].

To summarize, microRNA circadian regulation has been addressed in animal models, especially mice models. However, the knowledge about the circadian regulation of human microRNAs is still in its infancy and, thus, we must carry out more studies focusing on human circadian microRNAs to explore more cellular and tissue types. In addition, we need to know the role that circadian microRNA’s misregulation could play in human disease.

### 5.2. Micromanaging the Circadian Clock

The gene network that controls the circadian system is well described. However, like other genes, circadian genes are also post-transcriptionally regulated. Among the epigenetic mechanisms that regulate circadian genes expression, microRNAs are the least studied. However, the association between microRNAs and the circadian system has gained recent attention (Figure 2).

miR-219 plays an important role in the regulation of the circadian cycle speed. Experiments performed by Cheng *et al.* in murine models showed that silencing miR-219 using intracerebroventricular infusion of antisense oligonucleotides, produces prolongation in the circadian cycle. The underlying mechanism is unclear, although the regulation of miR-219 by *CLOCK* and *BMAL1* genes in PC12 cells has been reported [126]. miR-122, a well-studied liver specific microRNA, reduces hepatic Nocturnin expression, a deadenylase that has been implicated in post-transcriptional regulation of lipid metabolism and circadian clock. However, due to the long half-life of miR-122, this microRNA accumulates in the liver, questioning the real involvement of this microRNA in the short-term circadian cycle regulation [127].

Using a brain-specific *Dicer* knockout mouse model, Chen *et al.* described an important two-hour shortcut in circadian cycles of mutant mice. The explanation of this shortcut could be faster *Per1* and *Per2* translation, which are negatively regulated by miR-24, miR-29a, and miR-30 [128]. However, other experiments using hepatocyte-specific *Dicer* inactivation, failed to show core clock genes alterations, albeit they found widespread effects of hepatic microRNAs impairment on clock output gene expression. These results suggested that the hepatic clock is strongly resilient to microRNAs and that circadian system microRNAs-mediated regulation could be tissue-specific [129]. miR-24 is clustered with miR-23 and miR-27 and has been associated with T2DM, heart, and β-cell function [130]. miR-29 has also been associated with diabetes and its complications [131] and has been proposed as a potential circulating biomarker for T2DM and atherosclerosis [132]. miR-30c, a member of the miR-30 family, has been implicated in hepatic and plasma lipid regulation [133] and is modulated by docosahexanoic acid (DHA) in Caco-2 cells [134].

miR-185, a microRNA involved in cholesterol metabolism [135,136,137], and modulated by palmitic acid in HepG2 cells [138] decreases *Cry1* levels in murine models [139]. Using cell lines, Shende *et al.* demonstrated that miR-142-3p and miR-494 overexpression decreases endogenous *BMAL1* levels and increases *PER2* oscillations, suggesting that both microRNAs play an important role in post-transcriptional modulations of core molecular clockworks in this cell lines [140]. miR-142-3p is associated with the development, migration, and invasion of hepatocellular carcinoma via RAC1 protein levels and function regulation [141]. On the other hand, miR-494 is related to lung cancer [142], gastrointestinal cancer [143], brain tumor [144], and nasopharyngeal carcinoma [145]. Moreover, miR-494 has insulin-like growth factor 1 receptor (*IGF1R*) as a predicted target [146].

Our group demonstrated that miR-107 targets *CLOCK* in Caco-2 cells, a cellular model of human enterocytes. miR-107 oscillates in a circadian manner and miR-107 overexpression altered the circadian rhythm of these cells. Thus, miR-107 is a circadian microRNA that modulates the circadian system. Moreover, we demonstrated that miR-107 is modulated by dietary lipids both *in vitro* and *in vivo*. These results link circadian disruptions and diet with microRNAs [110]. miR-107 has long been recognized for its association with insulin sensitivity [109,147].

Finally, some of the circadian-related microRNAs have been found in plasma and could have a relevant role as biomarkers of circadian disruption. miR-494, miR-152, and miR-142-3p have been detected in mouse blood serum and were predicted to target 3′ UTR *BMAL1* mRNA [141,148,149]. All these results suggest the importance of circulating microRNAs in the regulation of peripheral circadian cycles.

To conclude, many microRNAs can control circadian genes expression. Many of them are also subjected to a circadian control and can be found in plasma. This means they could be potential pharmacological targets for circadian disorders. However, to this end, it is necessary to gain a deeper insight about the role of microRNAs in circadian regulation of cellular physiology and more evidence should be obtained from human studies.

## 6. Concluding Remarks

Although the importance of time for life in general has been recognized since the Greek and Roman civilizations, the discovery of the specific role of time in health and disease is relatively recent. During he past decade the research field of chronobiology has greatly expanded. However, we are far from the finish line. The circadian pathway is well known and the consequences of chornodisruption are well elucidated. However, the regulation of the circadian system in response to environmental inputs is currently being revealed and little is known about the role of variants in circadian-related genes in the individual predisposition to disease. Some variants in *CLOCK* and other related genes modify the individual risk of developing metabolic diseases, such as obesity or T2DM. Moreover, some gene-diet interactions have been described that modulate the individual predisposition defined by those variants. These findings are promising and encourage the use of chronotherapy from a nutrigenetic point of view in the frame of personalized nutrition. Chronotherapy can be implemented to manage sleep or mood disorders. Different approaches exist, including bright light therapy, regulation of sleep-awake cycles, or melatonin supplements [150]. Bright light therapy has been applied to depression [151], sleep problems [152] and Alzheimer’s Disease [153]. Regular sleep-awake cycles and controlled light exposure therapies have been applied to shift workers resulting in overall positive effects on chronic diseases [32]. Melatonin supplementation is a potential tool to manage circadian-related diabetes [154]. Instead of using melatonin supplements, the use of melatonin-rich diets linked to chronotherapy could be useful to prevent or even treat metabolic disorders. Additionally, chronotherapy could be applied to dietary interventions to lose weight, as suggested by the Garaulet’s population studies [5,6,155]. The introduction of genetic information to chronotherapy may further increase the expected chronotherapy effectiveness. However, most population studies analyzing circadian gene variants are composed by Caucasian participants and more studies are needed to know if those findings can be extended to other ethnic populations. Studies of other ethnic populations are scarce but Asian studies suggest that described variants are not relevant in disease risk in Asian individuals. Thus, other variants could exist that may modulate disease risk in populations other that Caucasians, but further research is needed in this regard. We should also bear in mind that most associations were nominally significant and lost significance after correction for multiple testing. Thus, before applying this knowledge, we should quantify the actual benefits of chronotherapy in personalized nutrition. As far as we know, there are not interventional studies assaying chronotherapy potential in personlized nutrition with the aim of weight or CVD risk reduction. Although the transcriptional/translational feedback loop that governs the circadian system is well known, little is known about how it is epigenetically regulated. Our knowledge about microRNA’s role in circadian regulation is in its infancy, but promising findings have been recently reported. It is worth mentioning that some circadian microRNAs can be modulated by diet which can be seen an effective tool to epigenetically modulate the circadian system in our fight against chronodisruption. Other features linking chronobiology and diet deserve to be highlighted, however they are not the focus of this review. This is the case of the relationship between microbioma, diet, and circadian rhythm, which add to the current complexity of this issue. The human microbioma displays diurnal rhythms and chronodisruption can lead to dysbiosis [156] and the microbiome is regulated by the host’s circadian clock. Conversely, the microbioma contributes to the dietary alterations of the host’s circadian clock [157]. The precise interplay between microbiome’s clock and host’s clock is unknown and further investigasion is necessary.

In conclusion, diet links chronobiology and human health at both genetic and epigenetic levels and this link is worth of further studies in order to include chronotherapy as part of personalized nutrition.

## Figures and Tables

**Table 1:** Summary of variants in circadian-related genes associated to obesity.

Gene	Nucleotide Polymorphisms (SNP)	Population	Association	Reference
Circadian locomotor output cycles kaput (CLOCK)	rs3749474	500 overweight/obese subjects from Spain (Caucasian) recruited into a weight-loss program	Carriers of the minor allele showed higher weight and body mass index (BMI)	[64]
		540 men and 560 women with overweight of the Genetics of Lipid Lowering Drugs and Diet Network population (GOLDN) population	Carriers of the minor allele showed higher energy intake	[65]
	rs1801260	500 overweight/obese subjects from Spain (Caucasian) recruited into a weight-loss program	Carriers of the minor allele showed higher weight and BMI,shorter sleep and lower response to a weight-loss program	[64]
		1495 overweight/obese subjects from Spain (Caucasian) recruited into a weight-loss program (82.5% females)	Carriers of the minor allele showed a lower response to a weight-loss program, especifically among those subjects characterized as ”emotional eaters”, had higher ghrelin plasma levels and showed an evening chronotype	[66,67]
		85 overweight caucasian women (43 women carrying the C allele and 42 women with the TT genotype)	Women carrying the C allele showed a tendency to the eveningness and less stable rhtyhms	[69]
		540 men and 560 women with overweight of the GOLDN population	Carriers of the minor allele showed higher waist circumference when saturated fatty acids (SFA) intake was ≥11.8%	[71]
		475 subjects with MetS from the CORDIOPREV study	Homozygote subjects for the major allele showed lower insulin levels, lower HOMA-IR (homesotatic model assesment-insulin resistance) and higher insulin sensitivity after 12 month of a low-fat diet	[72]
		772 participants of the Boston-Puerto Rican Health Study	Minor C allele was protective against high low-density lipoprotein (LDL)-cholesterol plasma levels in the group with high SFA intake	[73]
		2485 subjects enrolled in the baseline surveys of the Japan Multi-institutional Collaborative Cohort (J-MICC) Study	Minor C allele was associated with higher risk of prevalent diabetes, especially in lean subjects	[74]
	rs4580704	540 men and 560 women overweight of the GOLDN population	Carriers of the minor allele showed lower weight, BMI, blood pressure, postpandrial triglyceridemia and risk of type 2 diabetes mellitus (T2DM) and hypertension. A gene-diet interaction exists with monounsaturated fatty acids (MUFA) intake and T2DM-related parameters	[71]
		540 men and 560 women overweight of the GOLDN population	Carriers of the minor allele showed lower energy intake	[65]
		7098 subjects with high cardiovascular risk from the PREDIMED trial	The G allele was associated with lower weight, BMI, and waist circumference and with lower fasting glucose in non-diabetic subjects. The protective effect of the G allele againts T2DM was higher in the Mediterranean Diet intervention group	[75]
CRY1	rs2287161	728 Mediterranean (81% women) and 820 North American (50.5% women) overweight subjects	Carriers of the minor C allele showed higher HOMA-IR when carbohydrate intake was >41.65% of total energy	[76]
	rs12315175	Meta-analysis using 3 cohorts composed by participants of different ethnic origin	This variant is associated with a lower risk of T2DM	[82]
CRY2	rs11605924	3210 unrelated Chinese Hans from Beijing and Shanghai	This SNP has been assocaited with lower fasting glucose levels and risk of T2DM	[77]
		4654 non-diabetic Finland subjects from the PPP-Botnia Study.	This variant is associated with a better β-cell function	[80]
		721 obese individuals following a weight-loss program with diets with different fat and protein composition from the POUND LOSS trial (Harvard School of Public Health and Brigham and Women’s Hospital in Boston, MA, USA)	The variant allele was associated with a lower respiratory quotient and higher resting metabolic rate	[81]
		Meta-analysis including 28,190 participants of European descendent from CHARGE consortium	The previously reported association of this SNP and fasting glucose was not replicated in this meta-analysis	[78]
	rs2292912	Meta-analysis using 3 cohorts composed by participants of different ethnic origin	This variant is associated with a higher risk of T2DM	[82]
PER2	rs4663302	454 overweight/obese subjects from Spain (Caucasian) recruited into a weight-loss program	Homozygote carriers of the minor allele showed higher abdominal obesity and were prone to withdraw from a weight-reduction program	[84]
	rs2304672	454 overweight/obese subjects from Spain (Caucasian) recruited into a weight-loss program	Carriers of the G allele showed lower waist-to-hip ratio but they also showed eating behaviors alterations	[85]
	rs7602358	3512 subjects with from the Pujabi cohort resident in the United Kingdom and Pakistan	The G allele was negatively associated with T2DM	[82]
Brain and muscle ARNT-like protein 1 (BMAL1)	rs11022775	3512 subjects with from the Pujabi cohort resident in the United Kingdom and Pakistan	The T allele is associated with an increased risk of T2DM	[82]
	rs6486121	1304 individuals from 424 British T2D families from the Diabetes in Families (DIF) study collection	Variant allele is nominally associated with hypertension	[86]
	rs7950226	1304 individuals from 424 British T2D families from the Diabetes in Families (DIF) study collection	Variant allele is nominally associated with T2DM	[86]
REV-ERBα1	rs2314339	1402 Mediterranean (82% women) and 810 North American (48.2% women) overweight subjects	Carriers of the minor allele showed lower waist circumference, BMI and abdominal adiposity. An interaction with MUFA intake was found for BMI	[87]
	rs939347	1197 Spanish subjects, 779 of them obese	AA genotype was most frequent among men obese subjects	[88]
	rs2071427	3480 adolescents and adults from three independent population-based studies (MONICA, MONA-LISA and HELENA)	The minor T allele is associated with higher BMI in adults and adolescents	[89]
MTNR1B	rs1387153	GWAS data from 2151 nondiabetic French subjects	T allele was associated with higher fastin glucose levels and higher risk of developing hyperglycemia and T2DM	[90]
		Meta-analysis including 28,190 participants of European descendent from CHARGE consortium	T allele was associated with higher fastin glucose	[78]
		2222 Asian subjects (1201 T2DM and 1021 Control)	There was no association between this SNP and fasting glucose levels	[95]
	rs10830963	GWAS conducted in 6 cohorts of European origin	The risk allele was associated with higher fasting glucose, lower insulin secretion and also showed a weak association with the risk of developing T2DM	[91]
		10 GWAS involving a total of 36,610 individuals of European descent	G allele is associated with higher fasting glucose, lower β-cell function and the risk of T2DM	[92]
		346 Caucasians, 218 African-Americans, and 217 Hispanics obese children and adolescents	The MTNR1B rs10830963 variant was associated with higher fasting glucose levels and lower dynamic β-cell response in Caucasians and Hispanics	[93]
		Meta-analysis including 28,190 participants of European descendent from CHARGE consortium	G allele is associated with higher fasting glucose and HOMA-IR	[78]
		721 obese individuals following a weight-loss program with diets with different fat and protein composition from the POUND LOSS trial (USA)	Carriers of the G allele showed higher respiratory quotient in relation with the dietary fat	[81]
		A meta-analysis including 113,025 T2DM patients and 199,997 controls from 38 studies	There was an association between this SNP and T2DM risk in Caucasian but not Asian subjects	[94]
		2222 Asian subjects (1201 T2DM and 1021 Control)	There was no association between this SNP and fasting glucose levels	[95]
	rs1374645	2222 Asian subjects (1201 T2DM and 1021 Control)	This SNP was associated with lower fasting glucose in subjects with low BMI	[95]

## Abbreviations

MetS	Metabolic Syndrome
T2DM	type 2 Diabetes Mellitus
CVD	Cardiovascular Disease
SCN	Suprachiasmatic Nucleus
CCG	clock controlled genes
PUFAs	polyunsaturated fatty acids
SNPs	single nucleotide polymorphisms
SFA	saturated fat intake
MUFA	monounsaturated fatty acids
